# Paroxysmal Ventricular Standstill—A Case Report of all Ps and no QRS in Ventricular Asystole

**DOI:** 10.21980/J8SS79

**Published:** 2020-10-15

**Authors:** Hamid Ehsani-Nia, Christopher Bryczkowskiv

**Affiliations:** * Rutgers Robert Wood Johnson Medical School, Department of Emergency Medicine, New Brunswick, NJ

## Abstract

Paroxysmal ventricular standstill, also known as ventricular asystole, is a very rare, often fatal arrhythmia that should be included in the differential of patients presenting with syncope. 1 In this case, a 68-year-old female presented to the emergency department (ED) after sustaining a syncopal episode and subsequent cardiac arrest. On review of cardiac telemetry, she was found to be having episodes of ventricular standstill (VS). Due to her altered mental status, transcutaneous and then transvenous cardiac pacing were initiated. The patient was taken to the catheterization lab where she was found to have no significant coronary blockage, and a permanent pacemaker was placed. The likely culprit was calcium channel blocker toxicity. Paroxysmal ventricular standstill is a non-perfusable arrhythmia that should be rapidly identified. While VS is often a fatal arrhythmia presenting with sudden death, reversible causes should be pursued and treated.^2^

**Topics:**

Ventricular standstill, ventricular asystole, heart block, cardiac arrest, emergency pacemaker, transcutaneous pacemaker, transvenous pacemaker, cardiology, Visual EM.

**Figure:**
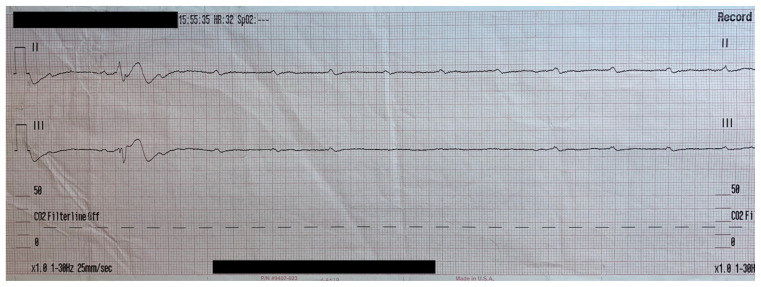
Obtained in the field prior to arrival.

**Figure:**
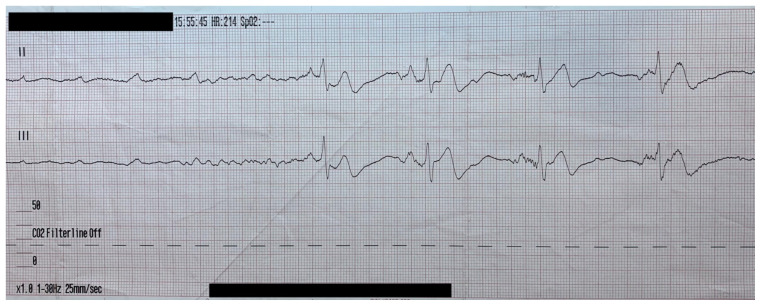
Obtained in the field prior to arrival.

**Figure:**
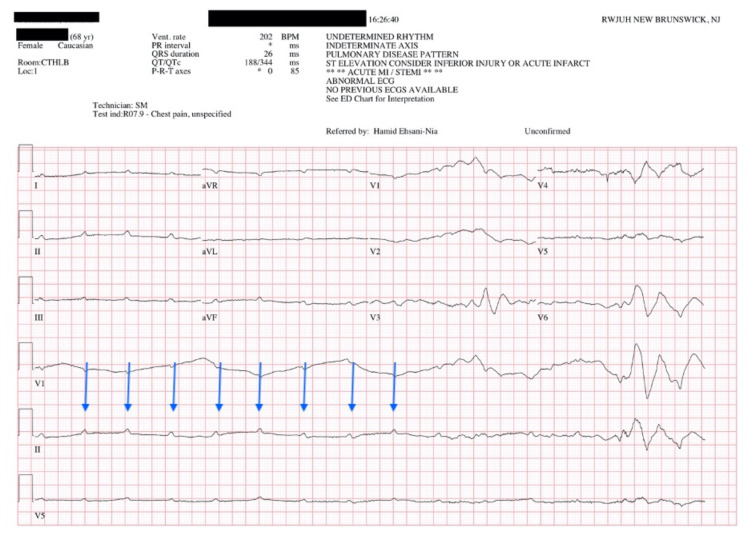
ECG obtained upon ED arrival. Transcutaneous pacemaker was paused while obtaining this ECG.

## Introduction

Ventricular standstill (VS) is a potentially fatal and rare arrhythmia which requires rapid identification and treatment. 3 As in ventricular fibrillation (VF), during VS there is a sudden loss of cardiac output. As a result, patients typically present with repeated episodes of syncope, although dizziness, sudden cardiac death and even seizures have been described. 3–5 In comparison to VF, the underlying mechanism is quite different. In VF, there is a lack of coordinated contractions causing the ventricles to quiver. In VS, there is a lack of impulse formation in the cardiac conduction system which in turn causes the heart to stop beating at all. 6,7 During such episodes, lack of awareness of VS as a potential etiology may lead to delays in definitive care. 3 Here, we present a case of a patient who had numerous syncopal episodes and subsequent cardiac arrest as a result of underlying VS.

## Presenting concerns and clinical findings

A 68-year-old female with past medical history of diabetes (treated with metformin), hypertension (treated with trandolapril and verapamil 200mg extended release once a day), and hyperlipidemia (treated with atorvastatin) presented with loss of consciousness while sitting on a bench, falling forward and striking her head. On advanced life support (ALS) arrival, the patient was found to be altered and developed brief, intermittent losses of conscious that spontaneously self-resolved within a few seconds. These episodes were associated with weakly palpable pulses (Rhythm Strip 1). In route to the emergency department (ED), these episodes became more frequent, longer lasting and became associated with repeated loss of consciousness and lack of palpable pulses. (Rhythm Strip 2). Cardiopulmonary resuscitation (CPR) was initiated for these episodes and return of spontaneous circulation (ROSC) was established in a matter of seconds each time.

ALS telecommunicated these rhythm strips from the field to the emergency physician provider providing on-line medical control which revealed an intermittent complete lack of cardiac ventricular activity. Due to patient’s unstable condition, orders were relayed for initiation of transcutaneous pacing as well as administration of intravenous (IV) fentanyl for pain control.

Upon arrival to the ED the patient was lethargic while being transcutaneously paced. Vital signs at that time included a blood pressure of 205/95, pulse at 80 beats per minute (intrinsic pulse of 36), respiratory rate of 12 and pulse oximetry at 88%. History obtained by bystanders as well as family relayed an episode of syncope, without prodrome, as well as adherence to her medications. Transcutaneous pacing was shortly paused in order to obtain a 12 Lead ECG in the resuscitation bay (ED ECG).

## Significant findings

In route, it was proposed that this patient was suffering from a dysrhythmia due to the transient episodes of syncope with lack of ventricular activity on telemetry. Upon close examination of the rhythm strips as well as the ECG, *P* waves can be visualized without any accompanying QRS complexes lasting multiple seconds (ED ECG blue arrows). Additionally, the rhythm has an intrinsic rate of 100 beats per minute and has a consistent morphology with no evidence of ventricular activity due to the lack of QRS complexes. As a result, the rhythm likely originates in the atria with no passage of impulses into the ventricles through the atrioventricular (AV) node versus an accelerated ventricular rhythm where QRS complexes would be seen.^8^ These rhythm strips demonstrate an example of VS. There is preserved native atrial automaticity, with an intact sinoatrial (SA) node, with a complete lack of ventricular electrical activity.

## Patient course

As the patient was altered and unstable, she was intubated for airway protection in the ED. The patient was treated with calcium gluconate for potential reversible causes of AV blockade, including electrolyte abnormalities such as hyperkalemia or iatrogenic causes such as medications. The patient was being treated for her hypertension with verapamil, a non-dihydropyridine calcium channel blocker, which has been reported to cause AV blockade.^9^ Intravenous calcium gluconate administration showed no apparent clinical improvement and a temporary transvenous pacemaker was subsequently floated from the right internal jugular vein as the AV block became resistant to transcutaneous pacing. After reliable mechanical and electric capture were obtained, the patient was taken to the catheterization lab and found to have no significant coronary artery disease with only a 50% stenosis of the left anterior descending artery found. In the electrophysiology lab, the patient had a permanent pacemaker placed. The patient had a four-day hospital course and declined rehabilitation afterwards. She was discharged home without any noted neurological sequelae.

## Discussion

While ventricular standstill is an uncommon presentation of syncope, it should be considered in those with recurrent episodes without a prodrome. In cases of cardiac syncope, VS along with high-grade AV block, complete heart block and other dysrhythmias have been a described as potential causes triggering syncope. This has been termed Stokes – Adams Syndrome, where a sudden loss of cardiac output leads to transient loss of consciousness.^10^,^11^

The likelihood of an AV block progressing to a dysrhythmia such as VS depends on its location. Typically, second degree AV block Mobitz type I (Wenckebach) is benign and does not have associated symptoms.^12^ This is due to an intermittent block within the AV node with *PR* intervals of progressively longer durations until a *P* wave is dropped. Conversely, second degree AV block Mobitz type II is indicative of disease below the AV node such as the His-Purkinje system and presents with a non-conducted *P* wave. 13 An advanced form of Mobitz type II, termed “high grade AV block” can present with multiple non-conducted *P* waves. Complete, or third-degree AV block, presents with total AV dissociation during which the *P* waves and *QRS* complexes occur independent of each other. Mobitz type II blocks and greater are an indication for permanent pacemaker placement due to risk of bradycardia and asystole.^14^ VS often occurs with such conduction blocks but can occur without them. 15

In many patients, episodes of VS are very short and self-resolving. Patients presenting to the ED may be asymptomatic or appear transiently confused, and outside of these episodes may have a normal ECG. Often times, the diagnosis is made from prehospital recordings and/or halter monitoring. 3 As a result, VS can be misdiagnosed as complete heart block or even epilepsy. 16,17 Failure to palpate a pulse during such events may lead to a delay in initiating CPR and/or cardiac pacing. 3

There are several etiologies that can lead to such heart blocks. Physical exam maneuvers causing increased vagal tone like carotid massage and tilt test can cause paroxysmal AV block and ventricular arrest 18,19 Acute or chronic ischemia is a common pathological cause of AV nodal blockade which needs to be immediately considered. Ischemic events have been described to account for up to 40% of cases of AV block. 20 Electrolyte aberrancies, such as hyperkalemia, are other important causes.^6^ Idiopathic degeneration can also occur. Autoimmune conditions such as Systemic Lupus Erythematosus, as well as connective tissue diseases like sarcoidosis and amyloidosis have also been documented causes of high-grade AV Block. 21,22 Infectious etiologies such as Lyme disease and Dengue have also been reported as the cause of ventricular asystole. 23,24

A potential cause of AV blockade and subsequent VS in this patient was iatrogenic, possibly from her use of verapamil, as previously mentioned.^9^ Of note, any toxicity involving calcium channel blockers, beta blockers, or digoxin are capable of producing a high-grade AV dissociation. 25

While this was a case of VS, it was treated as are other advanced heart blocks, with the use of calcium, transcutaneous and if necessary transvenous pacing as a bridge to definitive interventional and/or electrophysiological cardiac care. 6,22,26

While asystole is not treated with cardiac pacing, the decision to pace ventricular standstill has been supported by European resuscitation guidelines if lasting more than 3 seconds because it may respond to pacing. 27

Emergency physicians should consider ventricular standstill in those presenting with recurrent, transient loss of consciousness. The presence of regular atrial electrical activity (in the form of isolated *P* waves) without any ventricular activity should cause concern. Consider reversible causes of toxicity that can be addressed in the emergency department. Treat VS as a 2^nd^ degree Mobitz II AV block or higher with prioritization for immediate transcutaneous vs transvenous pacing, if so required. In this case, this patient who suffered repeated out-of-hospital cardiac arrest was found to have intermittent episodes of VS. Rapid diagnosis and management led to a good neurologic and functional outcome.

## Supplementary Information






