# Supplementary figures and images for: Paroxysmal Ventricular Standstill—A Case Report of all Ps and no QRS in Ventricular Asystole
Source: J Educ Teach Emerg Med. 2020 Oct 15;5(4):V25–30. doi: 10.21980/J8SS79 (PMC10332522; doi:10.21980/J8SS79)

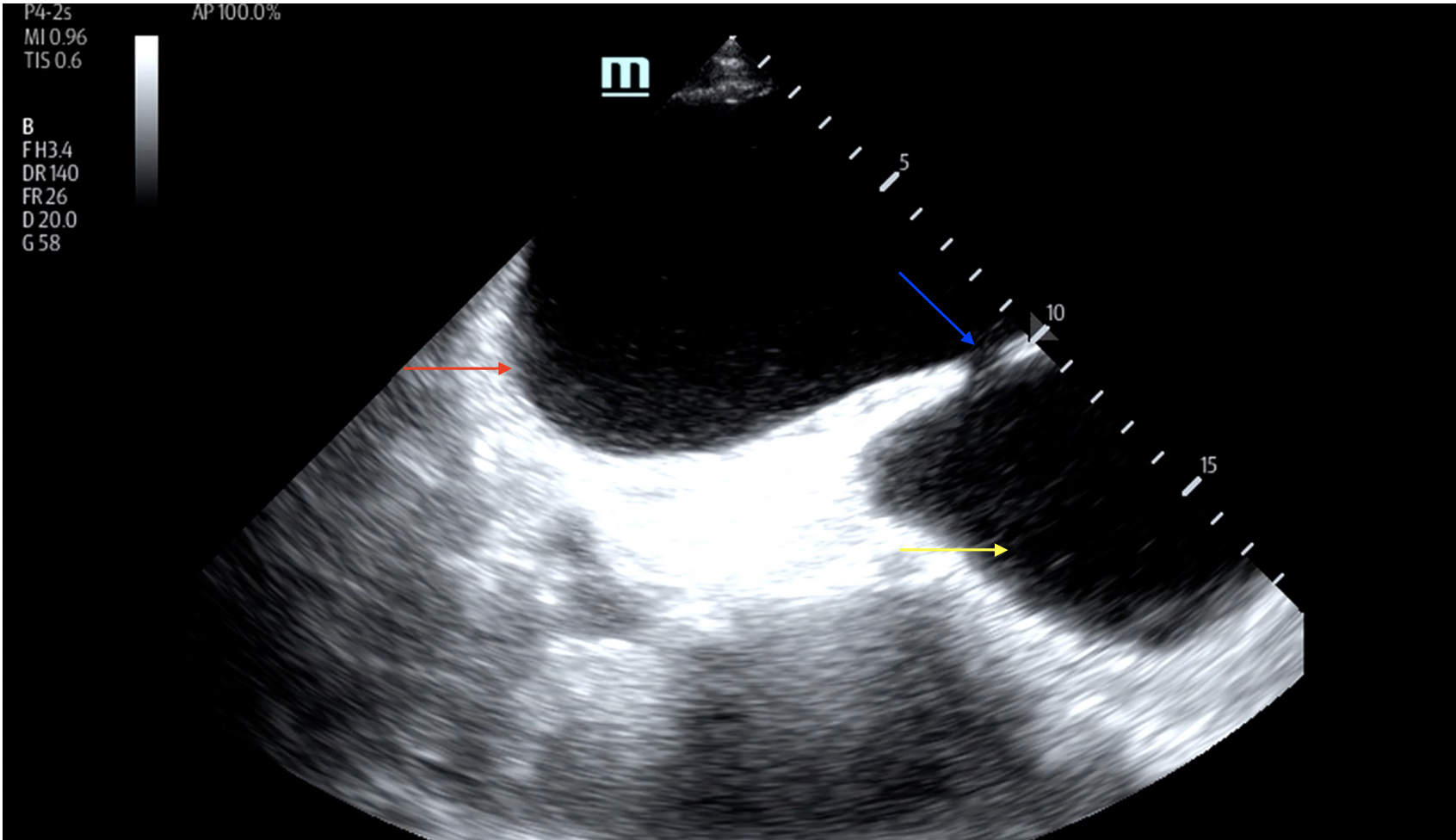

Supplement: Supplementary file 1 [file jetem-5-4-v15-supp1.jpg]

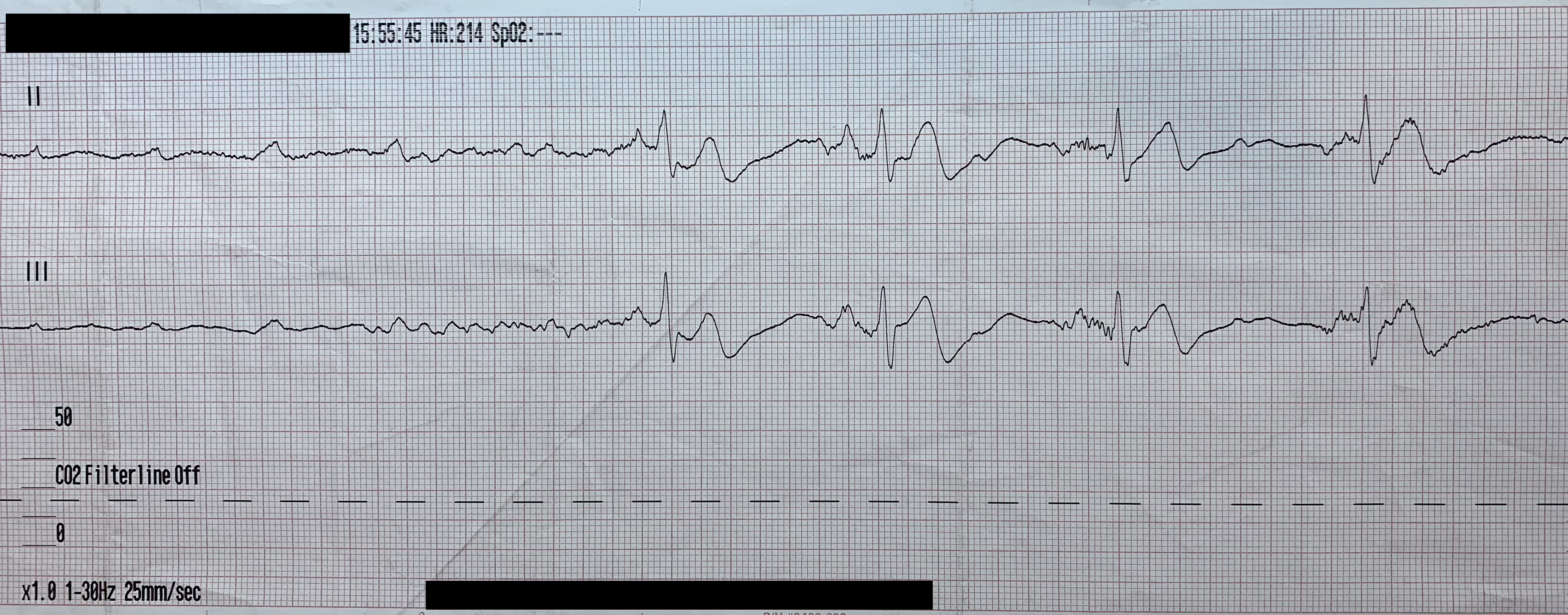

Supplement: Supplementary file 2 [file jetem-5-4-v25-supp2.jpg]

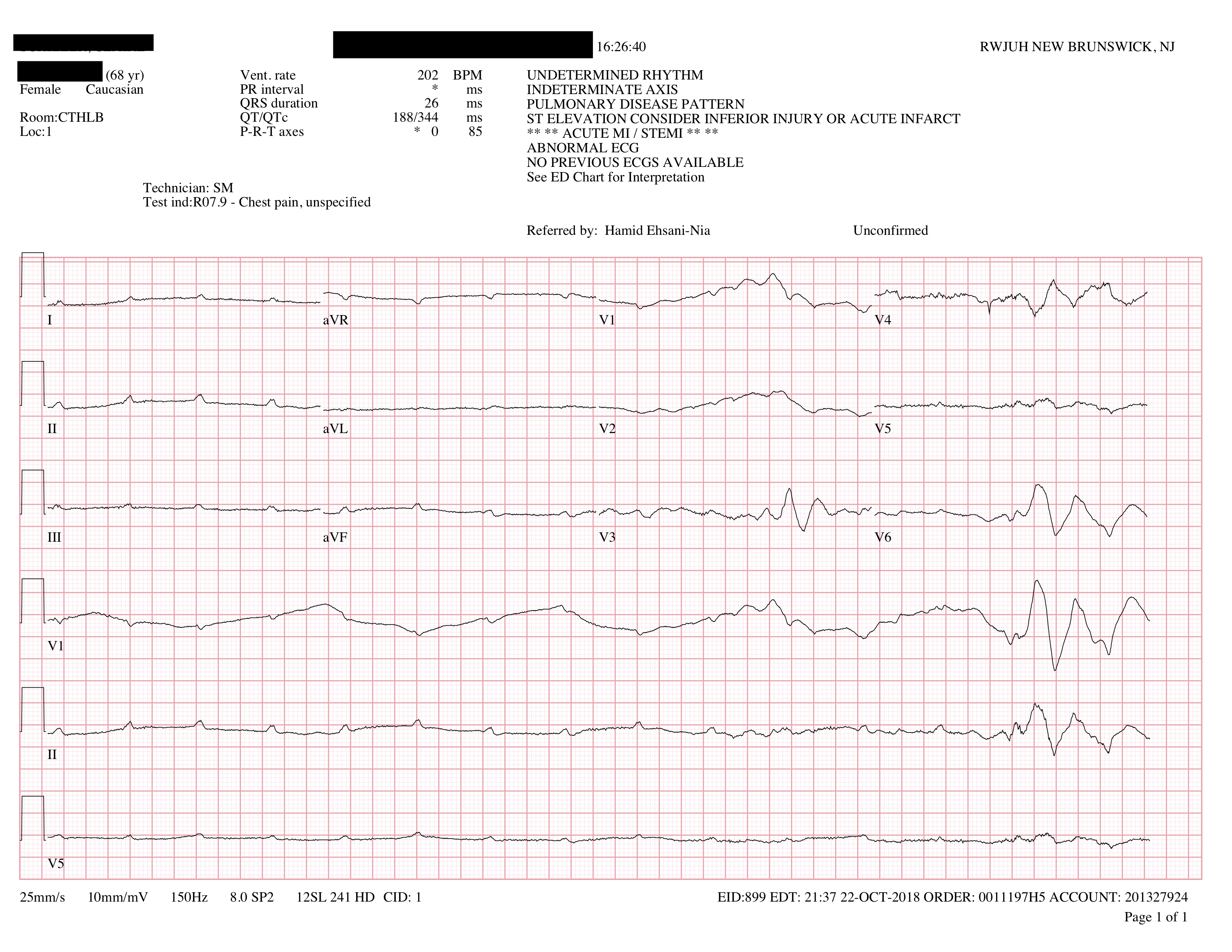

Supplement: Supplementary file 3 [file jetem-5-4-v25-supp3.jpg]
